# Repurposing of Milciclib as a Potential Inhibitor of *Toxoplasma gondii* in Human Trophoblasts and Placental Explants

**DOI:** 10.1021/acsomega.6c05747

**Published:** 2026-09-28

**Authors:** Rafael Martins de Oliveira, Natália Carine Lima dos Santos, Joed Pires de Lima Júnior, Luana Carvalho Luz, Marcos Paulo Oliveira Almeida, Guilherme de Souza, Izadora Santos Damasceno, Muriel Pereira Souto, Rosiane Nascimento Alves, Angelica Oliveira Gomes, Ingrid de Oliveira Dias, Juliana Quero Reimão, Bellisa Freitas Barbosa, Eloisa Amália Vieira Ferro, Samuel Cota Teixeira

**Affiliations:** † Laboratory of Immunophysiology of Reproduction, Institute of Biomedical Science, 28119 Universidade Federal de Uberlândia, Uberlândia 38400-694, Minas Gerais, Brazil; ‡ Department of Agricultural and Natural Science, Universidade do Estado de Minas Gerais, Ituiutaba 38302-192, Minas Gerais, Brazil; § Department of Clinical Medicine, Medicine College, Universidade Federal de Uberlândia, Uberlândia 38400-902, Minas Gerais, Brazil; ∥ Laboratory of Cell Interactions, Institute of Natural and Biological Sciences, Universidade Federal do Triângulo Mineiro, Uberaba 38025-350, Minas Gerais, Brazil; ⊥ Laboratory of Preclinical Assays and Research of Alternative Sources of Innovative Therapy for Toxoplasmosis and Other Sicknesses (Lab. PARASITTOS), Department of Morphology and Basic Pathology, 146840 Faculdade de Medicina de Jundiaí, Jundiaí 13202-550, São Paulo, Brazil

## Abstract

Congenital toxoplasmosis persists as a significant public health challenge due to the limited efficacy and safety of current treatments during pregnancy. In this study, we assessed the antiparasitic activity and mechanism of action of milciclib (MCB), a cyclin-dependent kinase inhibitor from the Medicines for Malaria Venture Pathogen Box, against *Toxoplasma gondii* using *in vitro* and *ex vivo* models of the maternal–fetal interface. MCB exhibited no cytotoxic effects in human trophoblastic BeWo cells or placental villous explants at concentrations up to 1.62 μM. The compound significantly reduced intracellular parasite proliferation in a dose-dependent manner, achieving approximately 39.72, 73.22, and 91.52% of inhibition at 0.40, 0.81, and 1.62 μM, respectively, and maintained a persistent antiproliferative effect following drug withdrawal. Importantly, MCB also reduced parasite burden in human placental explants (approximately 47.69% of inhibition) while preserving tissue viability and architecture. Mechanistically, MCB induced G1 phase arrest in *T. gondii* without affecting parasite adhesion or invasion, suggesting a specific action on intracellular replication. Ultrastructural analyses revealed marked morphological alterations in treated parasites, supporting a direct antiparasitic effect. In addition, MCB modulated host cell responses by reducing macrophage migration inhibitory factor (MIF) and reactive oxygen species (ROS) production accompanied by decrease in parasite burden, without triggering a pro-inflammatory profile. *In silico* analyses indicated potential interactions with parasite cyclin-dependent kinase-related proteins, particularly CRK2. Together, these findings identify MCB as a promising candidate for drug repositioning in congenital toxoplasmosis and support further investigation of its efficacy and safety *in vivo*.

## Graphical abstract

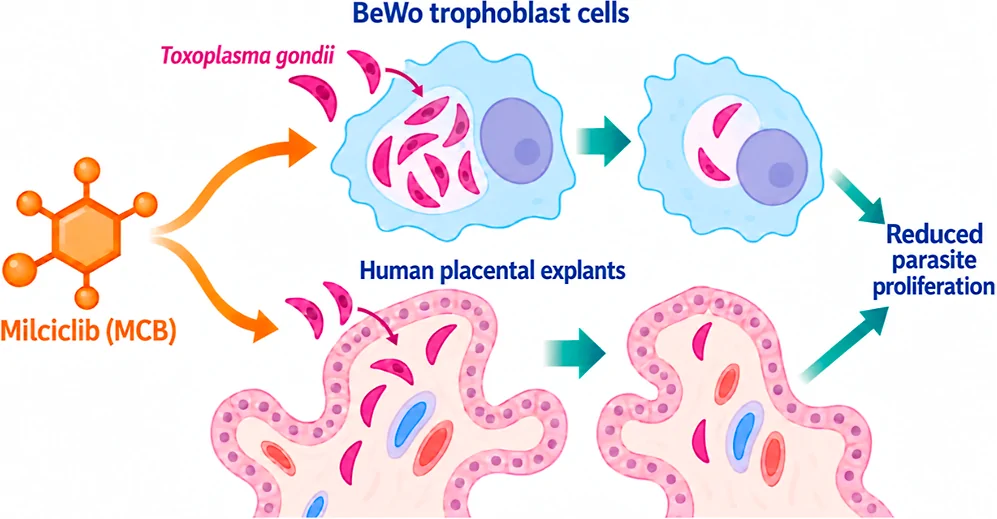

Toxoplasmosis is an infectious disease caused by the parasite *Toxoplasma gondii*, for which humans act as intermediate hosts. Transmission occurs primarily through the ingestion of contaminated water and undercooked or raw food, contact with soil contaminated with oocysts shed in the feces of infected cats, blood transfusion, and congenitally, from mother to fetus during pregnancy.^1,2^ Congenital toxoplasmosis represents the most severe form of infection, as vertical transmission may result in permanent fetal sequelae, particularly neurological impairments such as developmental delay, hydrocephalus, and encephalitis, as well as visual impairment.^3^ In more severe cases, this condition may lead to fetal death or prematurity.^4^ Therefore, early diagnosis and timely treatment are essential.

Conventional treatment of congenital toxoplasmosis involves a combined antiparasitic drug regimen. Spiramycin is administered in cases of maternal infection without confirmed fetal involvement.^5^ Following confirmation of fetal infection, a therapeutic regimen consisting of sulfadiazine and pyrimethamine, supplemented with folinic acid, is recommended.^6^ Although these drugs are effective, particularly during the acute phase of infection, they are associated with fetal side effects and potential teratogenicity,^5^ highlighting the need to explore alternative compounds that are both effective and well-tolerated for use during pregnancy.

In this context, bioactive compounds originally developed for other pathogens have been investigated as therapeutic strategies for toxoplasmosis. The Medicines for Malaria Venture (MMV) has established open-access compound libraries composed of a wide diversity of molecules with antiparasitic potential, such as the Pathogen Box. Several compounds from this collection have demonstrated anti-*T. gondii* activity in Vero cells,^7^ human trophoblastic BeWo cells, and in an *ex vivo* model using placental villi.^8^ These findings underscore the potential of MMV Pathogen Box compounds as promising sources of novel therapeutic strategies against toxoplasmosis. Based on this evidence, we expanded the investigation by evaluating another compound from this library, milciclib (MCB).

MCB is an experimental drug belonging to the class of cyclin-dependent kinase (CDK) inhibitors and has been widely evaluated in phase I and II clinical studies for the treatment of various tumor types.^9^ Its mechanism of action involves interference with G1 phase progression of the cell cycle and regulation of gene transcription, thereby limiting cell growth and proliferation.^10^ In Apicomplexan parasites such as *T. gondii*, cell cycle regulation is mediated by CDKs enzymes, which coordinately regulate the G1/S transition, DNA replication, and mitotic progression. Therefore, these proteins constitute attractive targets for therapeutic repurposing strategies.^11,12^ Although its properties have been primarily explored in the context of neoplastic diseases, MCB and similar CDK inhibitors compounds have been investigated in drug repositioning studies as a potential candidate for the treatment of neglected diseases caused by kinetoplastid protozoa, such as *Trypanosoma brucei*, *Trypanosoma cruzi*, and *Leishmania major*.^13^ However, to date, there are no reports in literature describing the experimental use in the treatment of congenital toxoplasmosis.

Despite considerable advances in understanding the biology of *T. gondii*, the treatment of congenital toxoplasmosis remains challenging due to the limited effectiveness and potential toxicity of the current available therapeutic options. In this sense, given the clinical impact of this disease and the limitations associated with currently available therapies, the investigation of novel molecules with efficacy against *T. gondii* and a favorable safety profile during pregnancy is highly warranted. Although MCB has shown promising parasiticidal activity in previous screening studies, no studies have evaluated MCB at the maternal–fetal interface. In this context, considering the documented antiparasitic activity and prior evidence supporting MCB as a candidate for drug repositioning, this study was designed to evaluate the anti-*T. gondii* activity of MCB and its potential mechanisms of action at the maternal–fetal interface using *in vitro* and *ex vivo* experimental models of BeWo trophoblast cells, a well-established model for investigating host–parasite interactions at the maternal–fetal interface, and human placental villous explants, which preserve the structural and cellular complexity of placental tissue, respectively.^8,14,15^

## Results

### Nontoxic Concentration of MCB Irreversibly Reduces *T. gondii* Proliferation and Induces Parasite Cell-Cycle Arrest

Initially, three different concentrations of the compound MCB were tested in BeWo cells, and cell viability was analyzed after 72 h of treatment using the MTT assay. The results showed that exposure to MCB (0.40, 0.81, and 1.62 μM) did not induce a significant cytotoxic effect on the cells, with viability remaining comparable to the control (RPMI medium only) across all concentrations evaluated. Additionally, treatment with PYR (1 μM) did not alter cell viability (Figure 1A).

**Figure 1:**
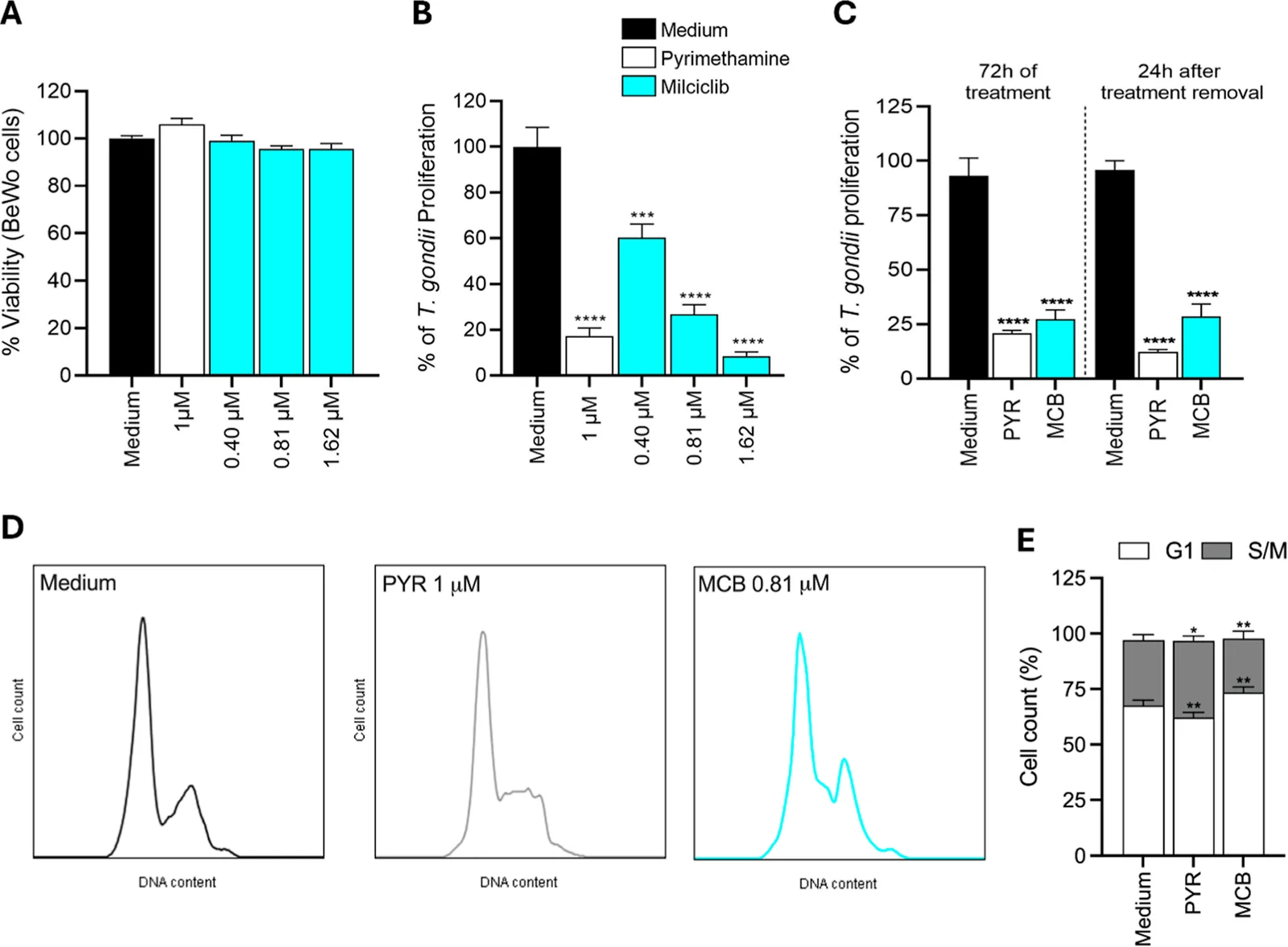
MCB’s low concentrations irreversibly control *T. gondii* proliferation in BeWo cells. (A) BeWo cells were cultured for 72 h in the presence of MCB (0.40, 0.81, and 1.62 μM) or PYR (1 μM). Cells maintained in culture medium alone were used as the positive viability control with viability defined as 100%. Cellular viability was evaluated through the MTT assay and presented as the mean percentage of viable BeWo cells. (B) *T. gondii*-infected BeWo cells treated for 72 h with MCB, PYR, or culture medium alone (defined as 100% parasite proliferation) following the same treatment scheme. Intracellular parasite replication was quantified through the β-galactosidase assay and presented as percentage change compared with the control (% *T. gondii* proliferation). (C) Infected BeWo cells were exposed for 72 h to MCB (0.81 μM), PYR (1 μM) or culture medium only; in a parallel condition, treatments were withdrawn after 72 h and cells were then maintained for subsequent 24 h drug-free period. At the end of each experimental condition, the intracellular parasite proliferation was evaluated through the β-galactosidase reaction. (D, E) Infected BeWo cells were treated for 72 h with MCB (0.81 μM), PYR (1 μM) or culture medium only. After the treatment period, intracellular parasites were recovered and fixed overnight in 70% ethanol at 4 °C. Parasites were fixed and incubated overnight with a mouse monoclonal antibody against *T. gondii*. After washing with PBS, samples were incubated with Alexa Fluor 488-conjugated antimouse IgG, RNase A (100 μg/mL), and PI (10 μg/mL), all diluted in PBS. Staining was performed for 45 min at room temperature in the dark. Finally, DNA content was analyzed using a flow cytometer. Data are represented as means ± standard error of the mean (SEM). *p < 0.05; **p < 0.01; ***p < 0.001; ****p < 0.0001 indicates statistical comparisons between untreated and treated cells. Significant differences were assessed using one-way ANOVA with Šídák’s multiple comparison posttest. A *p* value < 0.05 was considered statistically significant. Data are representative of at least three independent experiments, with each experiment including a minimum of eight replicates per group.

To assess the potential of MCB in controlling *T. gondii* proliferation in BeWo cells, we performed the β-galactosidase assay after 72 h of treatment. The results showed that the drug markedly reduced *T. gondii* proliferation in a concentration-dependent manner compared to the control (***p < 0.001, 0.40 μM; ****p < 0.0001, 0.81, and 1.62 μM; Figure 1B). As expected, PYR (1 μM) considerably decreased parasite proliferation following treatment (****p < 0.0001; Figure 1B). For the following experiments, we selected the intermediate concentration of MCB (0.81 μM), as it exhibited no toxicity to BeWo cells while effectively inhibiting intracellular *T. gondii* proliferation, thereby preserving cell viability without compromising antiparasitic efficacy.

To investigate the reversibility of MCB-induced antiproliferative activity, *T. gondii*-infected BeWo cells were treated for 72 h. Subsequently, the medium containing the compound was replaced with fresh medium, and the cells were maintained for an additional 24 h without treatment. Intracellular parasite proliferation was assessed using the β-galactosidase assay immediately following the 72 h treatment and after a subsequent 24 h drug-free period. The results showed that after 72 h of treatment, both MCB and PYR significantly inhibited parasite proliferation, and this effect persisted following drug removal (****p < 0.0001; Figure 1C), indicating a persistent impact on the replicative capacity of *T. gondii*.

Since MCB impaired parasite proliferation without affecting host cell viability, we investigated whether this effect could be associated with alterations in the parasite cell cycle. The results showed that PYR treatment led to a significant increase in the proportion of parasites in S/M (*p < 0.05), accompanied by a reduction in the G1 fraction (**p < 0.01) compared with the control (Figure 1D,E), whereas MCB treatment resulted in arrest of parasites in G1 phase and a decrease in the S/M population (**p < 0.01; Figure 1D,E), indicating that both drugs interfere with the replicative dynamics of *T. gondii*.

### Pretreatment with MCB Does Not Affect Early Steps of *T. gondii* Infection in BeWo Cells

To investigate whether MCB acted directly on the parasite or predominantly on the host cell to control parasitism in BeWo cells, we initially evaluated the effect of pretreating *T. gondii* with MCB or PYR for 1 h. The 1 h exposure period was chosen as the minimum time required to elicit measurable effects on tachyzoites without compromising parasite viability. Following treatment, parasites were incubated for 3 h with BeWo cells fixed or not with 4% PFA, for analysis of adhesion or invasion, respectively. After incubation, β-galactosidase activity was quantified. The results showed that, relative to the control, neither parasite adhesion to BeWo cells nor invasion was significantly altered after direct exposure of *T. gondii* to MCB or PYR (Figure 2A).

**Figure 2:**
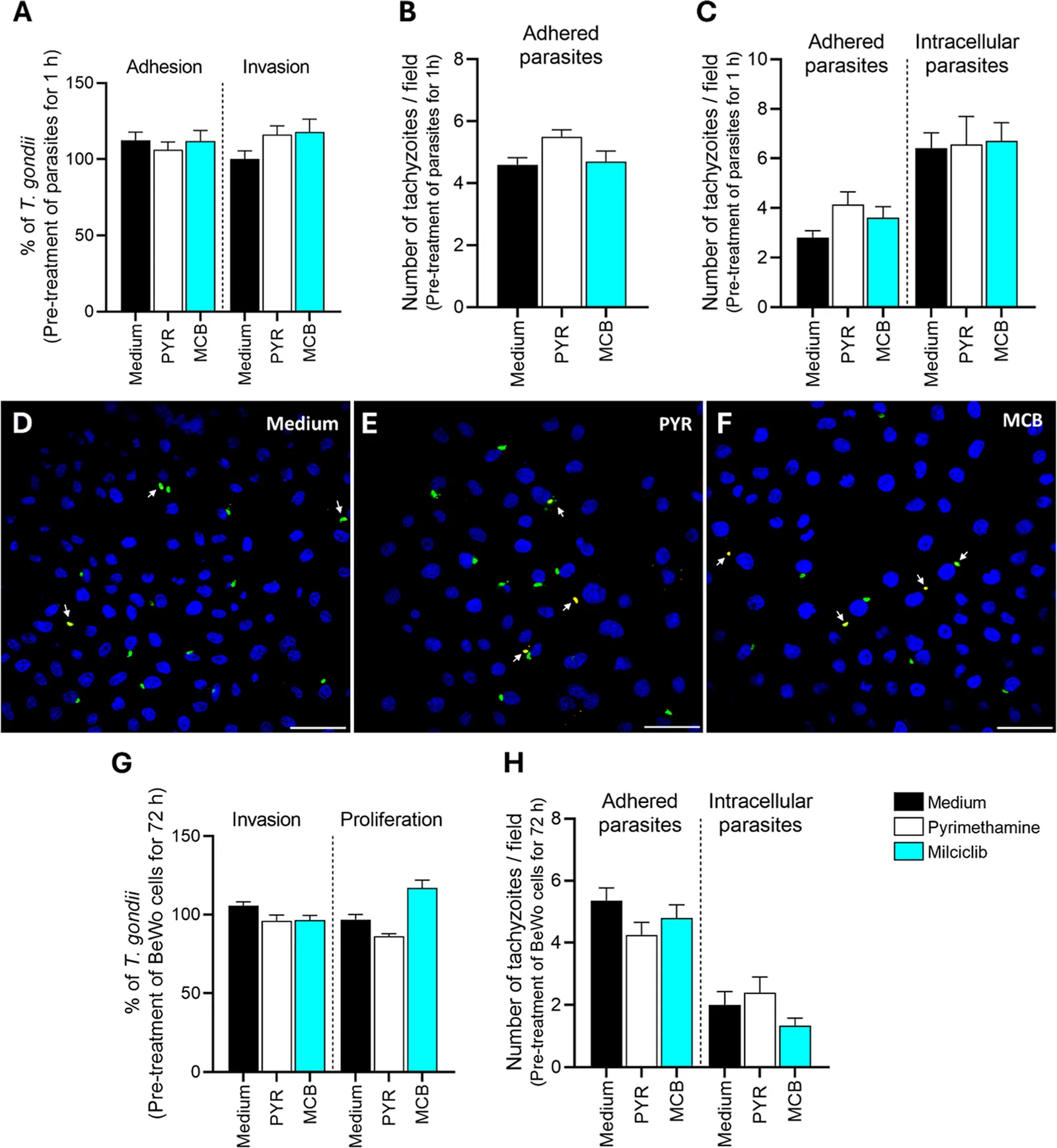
MCB does not interfere with early stages of *T. gondii* infection in BeWo cells. (A) Parasites were pretreated for 1 h with MCB (0.81 μM), PYR (1 μM), or culture medium only, and then incubated for 3 h with BeWo cells fixed or not for analysis of adhesion or invasion, respectively. Tachyzoites was measured through the β-galactosidase reaction and presented as a percentage of *T. gondii* adhesion or invasion. (B) Parasites were pretreated as described above and incubated with BeWo cells fixed on glass coverslips for 3 h. Next, samples were labeled with TO-PRO-3 iodide (nuclei), tetramethylrhodamine isothiocyanate (TRITC)-conjugated phalloidin (F-actin) and Alexa Fluor 488-conjugated antirabbit IgG (tachyzoites). Images were obtained using a confocal fluorescence microscope (Zeiss, LSM 510 Meta, Germany) with excitation wavelengths at 488 nm (argon laser) for Alexa Fluor 488, 543 nm HeNe laser for TRITC, and 633 nm (HeNe laser) for TO-PRO-3. Emission detection wavelengths were adjusted to 519 nm for Alexa Fluor 488, 570 nm for TRITC, and 661 nm for TO-PRO-3. An estimated of 20 images were randomly analyzed to acquire the total number of attached parasites per field. The results were presented as the ratio (number of tachyzoites/field). (C) *T. gondii* tachyzoites were exposed to MCB (0.81 μM), PYR (1 μM) or culture medium only for 1 h and then incubated for 3 h with BeWo cells on glass coverslips. Subsequently, parasites and host cells were stained with TO-PRO-3 Iodide (nuclei), Alexa Fluor 488-conjugated antirabbit IgG (internalized parasites) and Alexa Fluor 594-conjugated antirabbit IgG (adhered parasites). Images were obtained by confocal fluorescence microscopy with excitation wavelengths at 488 nm (argon laser) for Alexa Fluor 488, 543 nm HeNe laser for Alexa Fluor 594, and 633 nm (HeNe laser) for TO-PRO-3. Emission detection wavelengths were set to 519 nm for Alexa Fluor 488, 570 nm for Alexa Fluor 594, and 661 nm for TO-PRO-3. The number of intracellular (green^+^/red^–^) and adhered [red or red^+^/green^+^ (yellow)] tachyzoites were quantified on an estimated of 20 randomly selected fields on each coverslip. (D–F) Representative confocal images are shown, according to each experimental situation. White arrows indicate parasites adhered to the cells [red or red^+^/green^+^ (yellow)]. Scale bars: 50 μm. (G) BeWo cells were incubated for 72 h with MCB (0.81 μM), PYR (1 μM) or culture medium alone and then infected by *T. gondii* tachyzoites for 3 or 72 h for analysis of invasion or proliferation, respectively. The number of parasites was measured through the β-galactosidase assay and presented as a percentage of *T. gondii* invasion or proliferation. (H) BeWo cells were seeded onto glass coverslips and exposed to MCB (0.81 μM), PYR (1 μM) or culture medium alone for 72 h. Subsequently, the cells were incubated with *T. gondii* tachyzoites for 3 h. Next, cells and parasites were labeled with TO-PRO-3 Iodide (nuclei), Alexa Fluor 488-conjugated antirabbit IgG (internalized parasites) and Alexa Fluor 594-conjugated antirabbit IgG (adhered parasites). Images were acquired using the same procedure described above. The number of intracellular (green^+^/red^–^) and adhered [red or red^+^/green^+^ (yellow)] parasites was measured on about 20 randomly selected fields on each coverslip. Data are shown as means ± standard error of the mean (SEM). Significant differences were assessed using one-way ANOVA with Šídák’s multiple comparison posttest. A *p* value < 0.05 was considered statistically significant. Data are representative of at least three independent experiments.

We next employed a complementary approach based on immunofluorescence assays^16,17^ to directly evaluate parasite adhesion and invasion, allowing visualization and quantification of both cell–surface-associated and internalized parasites, thereby providing additional evidence regarding the impact of MCB treatment on the early stages of infection. For this, tachyzoites were pretreated for 1 h with MCB or PYR and subsequently incubated for 3 h with BeWo cells fixed, or not, with 4% PFA. Parasites and host cells were labeled and analyzed by confocal microscopy. The results showed that the adhesion capacity of tachyzoites to BeWo cells remained unchanged among the groups, as evidenced by both the quantification of adherent parasites (Figure 2B) and the red–green assay (Figure 2C), which distinguished internalized parasites from those attached to the cell surface. Representative images further confirmed that neither MCB nor PYR affected these early infection events (Figure 2D–F).

Subsequently, we assessed whether host cell pretreatment altered parasite establishment. For this, BeWo cells were treated with MCB or PYR for 72 h prior to incubation with *T. gondii* for 3 or 72 h to assess invasion or proliferation, respectively. Following infection, β-galactosidase activity was measured. The results demonstrated that neither parasite invasion nor intracellular proliferation differed from the control after host cell pretreatment with MCB or PYR (Figure 2G).

To further validate these observations, we performed the green–red assay to quantify internalized and cell-associated parasites following 72 h of host cell conditioning. Similarly, the results showed that the numbers of adherent and internalized tachyzoites did not differ significantly among the groups (Figure 2H).

Collectively, these findings demonstrate that although MCB efficiently controls *T. gondii* proliferation in BeWo cells, the initial steps of the parasite lytic cycle are not compromised when either tachyzoites or host cells are exposed to the drug prior to infection.

### MCB Compromises the Ultrastructural Integrity of *T. gondii* Tachyzoites

To elucidate the direct effects of MCB on *T. gondii*, tachyzoites-infected BeWo cells were exposed to MCB or PYR for 72 h. Following treatment, the parasite ultrastructure was analyzed by transmission electron microscopy (TEM). The images obtained showed that no significant morphological alterations were observed in the control group. The parasites presented preserved cellular architecture, with defined contours, homogeneous cytoplasm, and visible typical organelles, such as rhoptries (Rp) and dense granules (Dg). In addition, the parasite nucleus was well-defined, and the formation of the parasitophorous vacuole (PV) was clear and well-demarcated, compatible with metabolically active and structurally intact parasites (Figure 3A). In contrast, parasites exposed to PYR exhibited evidence of structural damage and loss of typical shape. Among the abnormalities observed were membrane deterioration, loss of definition and disorganization of organelles, as well as cytoplasmic vacuolization (Figure 3B). Treatment with MCB also promoted alterations in the structure of tachyzoites. Compared with the control, parasites treated with the drug exhibited an abnormal appearance, with contracted cell volume and undefined organelles. In addition, the PV was dilated, indicating a loss of the microenvironment necessary for parasite maintenance (Figure 3C). These results demonstrate that MCB compromises the ultrastructural integrity of *T. gondii*, in contrast to the preserved phenotype observed in the control, indicating an ability to directly interfere with parasite viability.

**Figure 3:**
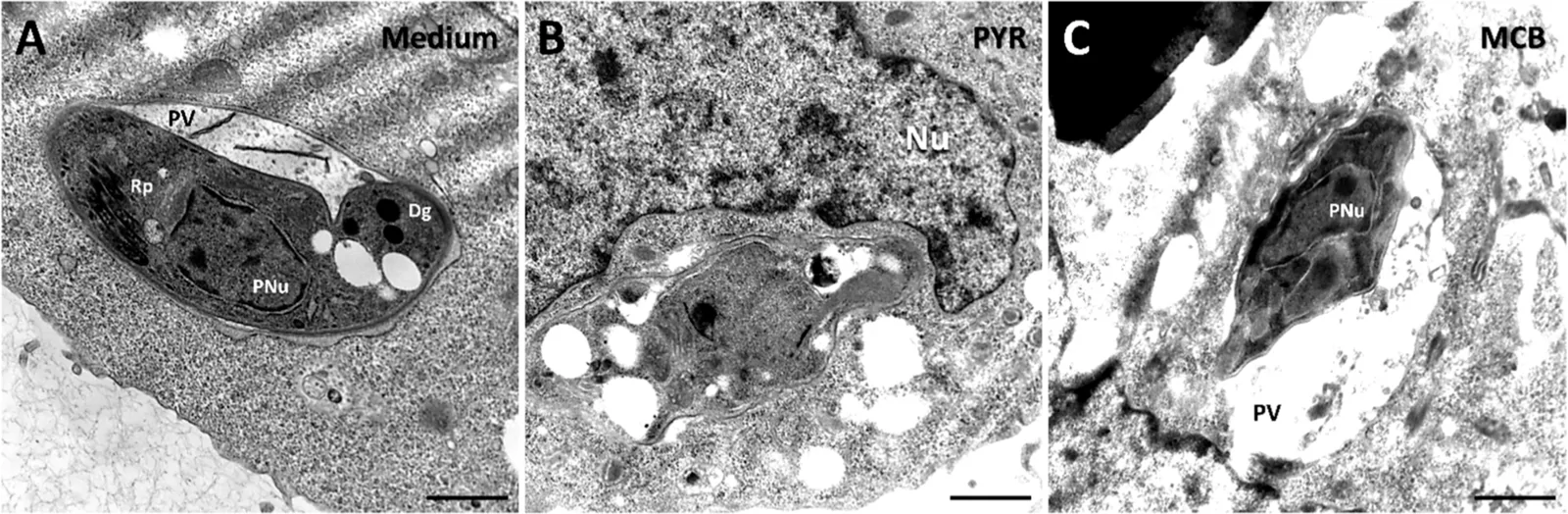
MCB promotes ultrastructural changes in *T. gondii*. BeWo cells were infected with *T. gondii* tachyzoites (MOI of 1:1) for 3 h and subsequently maintained for 72 h in the presence of MCB (0.81 μM), PYR (1 μM), or culture medium alone (untreated control). Transmission electron microscopy was performed using an accelerating voltage of 50 kV with images captured at a magnification of 12,000×. Uniform adjustments of brightness and contrast were applied to all groups to allow consistent visualization of fine structural features. Representative micrographs are shown according to the experimental group: (A) untreated cells, (B) PYR-treated cells, and (C) MCB-treated cells. Scale bars (bottom right): 1 μm. Dg, dense granule; Nu, host nucleus; PNu, parasite nucleus; PV, parasitophorous vacuole; Rp, rhoptries. Data are representative of at least three independent experiments, each carried out with a minimum of eight replicates per group.

### MCB Reduces MIF and ROS in Response to *T. gondii* Infection

To assess the immunoregulatory activity of MCB, we analyzed the production of IL-6, IL-8, and MIF in the absence and presence of *T. gondii* infection after 72 h of treatment with MCB or PYR. We observed that in uninfected cells, IL-6, IL-8, and MIF levels remained similar among groups. On the other hand, parasite infection promoted an increase in MIF production compared to uninfected control (^$$^ *p* < 0.01, Figure 4C). Regarding IL-8, the presence of *T. gondii* led to a significant increase in this cytokine in BeWo cells treated with PYR (****p < 0.0001, Figure 4B) or MCB (*p < 0.05, Figure 4B), compared to the infected control. Notably, MIF production decreased in infected cells following treatment with PYR (*p < 0.05, Figure 4C) or MCB (**p < 0.01, Figure 4C).

**Figure 4:**
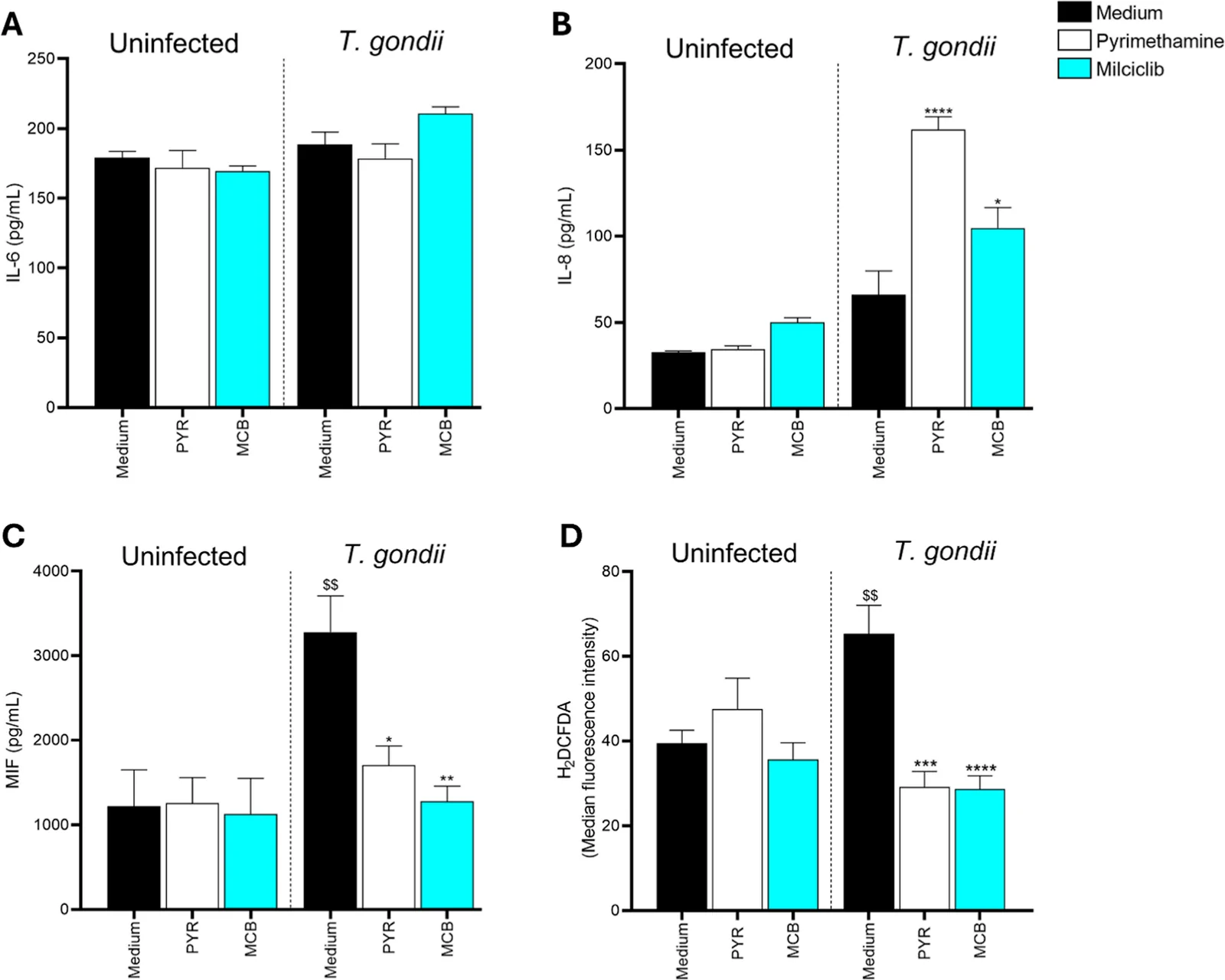
MCB downregulates MIF and modifies ROS production in response to *T. gondii* in BeWo cells. BeWo cells were infected with *T. gondii* tachyzoites or maintained uninfected and subsequently treated for 72 h with MCB (0.81 μM), PYR (1 μM) or culture medium (untreated group). Culture supernatants were collected for quantification of (A) IL-6, (B) IL-8, and (C) MIF, with cytokine concentrations expressed in pg/mL. (D) ROS production in BeWo cells was evaluated using the fluorescent probe H_2_DCF-DA. Data are presented as means ± standard error of the mean (SEM). *p < 0.05; **p < 0.01; ****p < 0.0001 indicates comparison between infected/untreated and infected/treated cell supernatants (A–C); ***p < 0.001; ****p < 0.0001 indicates comparison between infected/untreated and infected/treated cells (D); ^$$^ *p* < 0.01 indicates comparison between uninfected/untreated and infected/untreated supernatants/cells (C, D). Significant differences were assessed using one-way ANOVA with Šídák’s multiple comparison posttest. A *p* value < 0.05 was considered statistically significant. Data are representative of at least three independent experiments, with eight replicates per group.

We also evaluated whether MCB treatment would influence ROS production and observed no changes among groups in uninfected cells. In contrast, *T. gondii* infection triggered an enhance in ROS production in comparison to the uninfected control (^$$^ *p* < 0.01, Figure 4D), and treatment with PYR or MCB promoted a decrease relative to the infected control (***p < 0.001|****p < 0.0001, respectively; Figure 4D).

Collectively, these results indicate that MCB does not activate inflammatory responses under basal conditions, but during infection, modulates host cell immune responses by reducing inflammatory mediators and *T. gondii*-induced oxidative stress. However, whether this reduction reflects beneficial control of excessive inflammation or unintended immunosuppression that could impact parasite clearance requires further investigation.

### MCB Controls *T. gondii* Proliferation in Human Placental Explants

Human placental villi are *ex vivo* models extensively used to investigate *T. gondii* infection at the maternal–fetal interface. Therefore, we used MTT and LDH assays to evaluate the viability of villous explants after treatment with MCB or PYR for 72 h. The results demonstrated that MCB and PYR did not exhibit toxicity to placental explants compared to the untreated control (Figure 5A,B).

**Figure 5:**
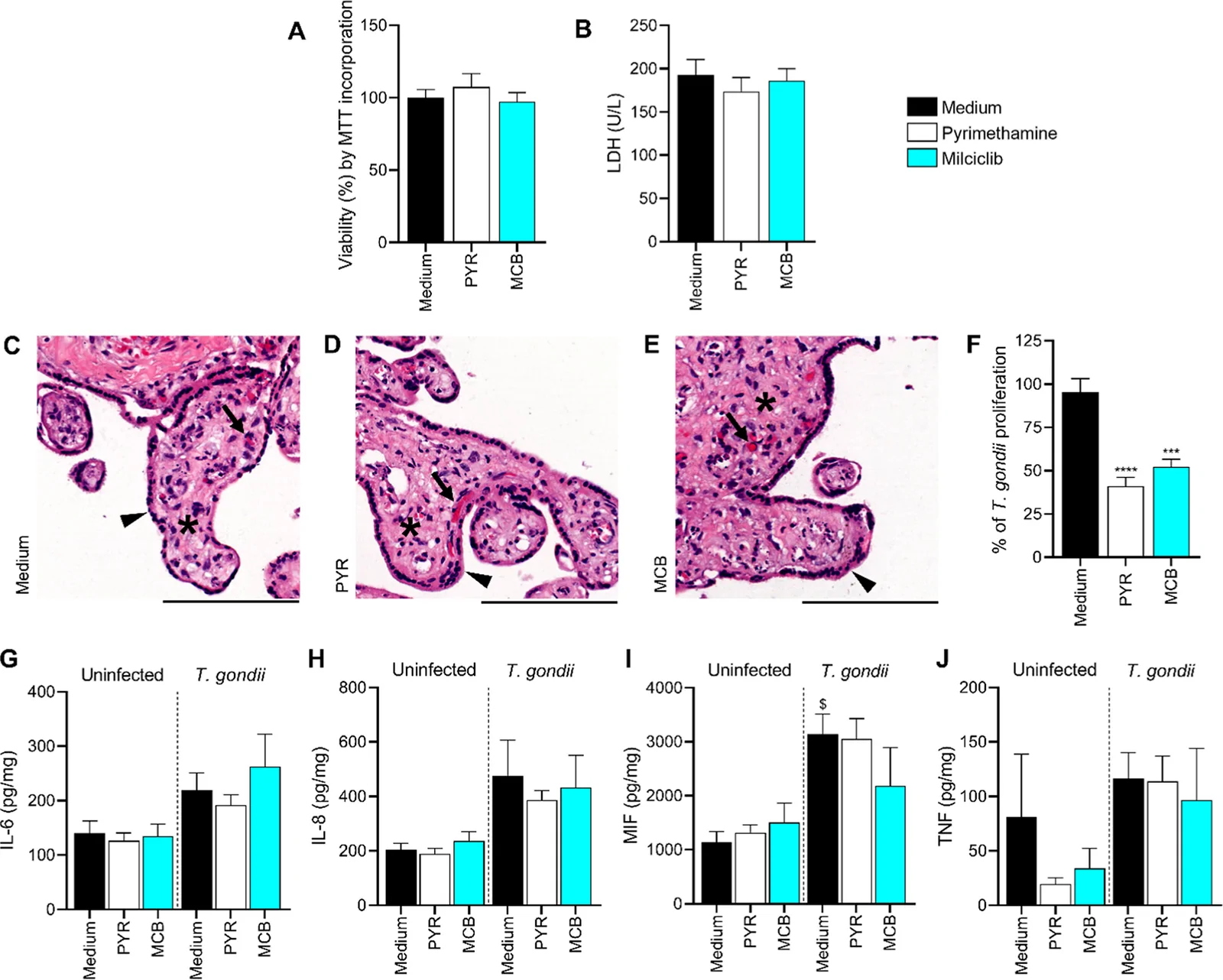
MCB reduces *T. gondii* proliferation in human placental villi. Placental explants were exposed for 72 h to MCB (0.81 μM), PYR (1 μM), or culture medium alone (untreated control), and then evaluated for tissue viability. (A) Explant viability was quantified through the MTT assay and presented as the mean percentage (%) of viable explants, with untreated villi defined as 100% viable. (B) LDH activity (U/L) was measured in the collected explant supernatants. Histological assessment was performed on HE-stained sections. Representative images at 10× magnification (scale bar = 200 μm) are shown, based to the experimental conditions: (C) untreated explants, (D) PYR-treated explants, (E) MCB-treated explants. (F) Placental villi were infected with *T. gondii* tachyzoites for 24 h and subsequently treated for 72 h with MCB (0.81 μM), PYR (1 μM), or culture medium alone (untreated control). *T. gondii* proliferation was determined through the β-galactosidase assay and expressed as percentage *T. gondii* proliferation, with the infected/untreated control considered as 100% of parasite proliferation. Supernatants from treated and untreated explants—infected or uninfected—were also analyzed for (G) IL-6, (H) IL-8, (I) MIF, and (J) TNF, reported as pg/mg of tissue. Data are presented as means ± standard error of the mean (SEM). ***p < 0.001; ****p < 0.0001 indicates comparison between untreated and treated villi (F); ^$$^ *p* < 0.01 indicates comparison between uninfected/untreated and infected/untreated supernatants (I). Significant differences were assessed using one-way ANOVA with Šídák’s multiple comparison posttest. A *p* value < 0.05 was considered statistically significant. Arrowheads indicate the outer multinucleated syncytiotrophoblast layer. Asterisks denote the mesenchymal core, enclosed by the syncytiotrophoblast and forming the internal architecture of the placental explant. Black arrows indicate fetal blood vessels surrounded by mesenchymal tissue and containing preserved fetal blood cells, predominantly fetal erythrocytes with characteristic eosinophilic staining. Data are representative of at least three independent experiments, each carried out with a minimum of eight replicates per group.

To support our observations, we stained the placental tissue with hematoxylin and eosin in order to analyze its structural integrity. Consistent with the biochemical data, we observed that treatment with MCB or PYR did not cause damage to the explants. Villi exposed to MCB or PYR maintained morphological characteristics equivalent to those observed in the untreated control, including the preservation of the multinucleated syncytiotrophoblast layer in the outer region (arrowheads) and a cellularized mesenchymal stroma (asterisks) containing intact fetal blood cells (black arrows) (Figure 5C–E).

Next, we used the β-galactosidase assay to assess the potential of MCB to control *T. gondii* infection in explants after 72 h of treatment. The results showed that PYR and MCB markedly decreased parasite proliferation compared to the untreated control (****p < 0.0001, PYR|***p < 0.001, MCB; Figure 5F).

We also quantified the cytokines released in the supernatants of placental villus cultures, in the absence or presence of *T. gondii* infection. The data showed that the IL-6, IL-8, MIF, and TNF levels remained similar among groups in both uninfected and parasite-infected explants (Figure 5G–J). In contrast, *T. gondii* infection led to an enhance in MIF expression in comparison to the uninfected control (^$^ *p* < 0.05, Figure 5I).

Together, these results show that MCB is well tolerated by placental villi at the tested concentration and shows promise in reducing the parasite burden and preserving tissue structure without exacerbating the inflammatory response.

### Identification of CDK-like Targets in *T. gondii*

BLASTp analyses identified three CDK-related kinases in *T. gondii*: CRK1, CRK2, and CRK3. All candidates presented significant alignment scores and conserved regions within the kinase catalytic domain, supporting their functional relevance. CRK2 was prioritized due to its compact structure and strong alignment within the catalytic domain, while CRK1 and CRK3 presented larger and more complex architectures (Supporting Table 1).

### Molecular Docking Analysis

Docking simulations demonstrated that MCB binds to all evaluated targets, with estimated binding free energies (Δ*G*) ranging from −7.22 to −8.45 kcal/mol. CRK2 exhibited the most favorable binding energy (Δ*G* = −8.45 kcal/mol), with the ligand positioned within the catalytic site. CRK1 also showed binding within the catalytic region (Δ*G* = −7.22 kcal/mol), whereas CRK3 displayed intermediate binding energy (Δ*G* = −7.64 kcal/mol) without a clearly defined catalytic localization. For human kinases, MCB binds preferentially to the ATP-binding pocket, with Δ*G* values of −7.33 kcal/mol for CDK1 and −7.41 kcal/mol for CDK4 (Supporting Table 2). Overall, these results suggest that MCB interacts with both parasite and human kinases with comparable binding energies. However, differences in binding site localization and structural context may contribute to differential target engagement.

### Structural Validation of CRK2

CRK2 was selected for further structural validation due to its favorable docking profile. Domain analysis (Pfam/InterPro) indicated that the protein is predominantly composed of a kinase domain, consistent with canonical CDK architecture (Supporting Figure 1). The three-dimensional structure of CRK2 was obtained from AlphaFold predictions, focusing on the kinase domain (∼300 amino acids). The model presented high confidence scores (average pLDDT ≈ 85), particularly within the catalytic region. Structurally, CRK2 exhibits the typical fold of serine/threonine kinases, including well-defined α-helices and β-sheets, supporting its functional annotation (Figure 6).

**Figure 6:**
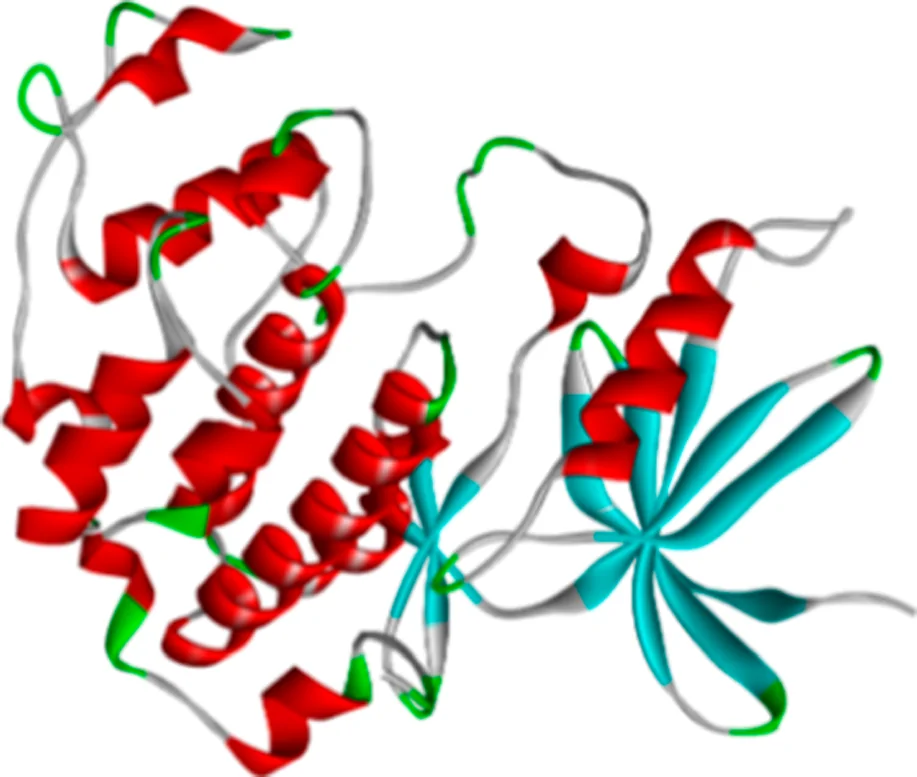
Structural model of the kinase domain of CRK2 from *T. gondii*. The three-dimensional structure of the CRK2 kinase domain was predicted using AlphaFold. The model highlights the typical kinase fold, composed of α-helices and β-sheets, with high-confidence regions (average pLDDT ≈ 85) predominantly localized within the catalytic domain. The structure supports the classification of CRK2 as a serine/threonine kinase and its potential role in parasite cell cycle regulation.

### Docking Analysis of MCB and CRK2

The docking of MCB to CRK2 revealed a stable interaction within the catalytic pocket, corresponding to the ATP-binding site. The best-ranked cluster showed a Δ*G* value of −8.45 kcal/mol. The ligand is positioned within a buried pocket formed by α-helices and β-sheets, indicating a stable interaction within the protein core rather than solvent-exposed regions. This binding mode is consistent with a potential ATP-competitive inhibition mechanism, similar to that observed for MCB in human CDKs (Figure 7).

**Figure 7:**
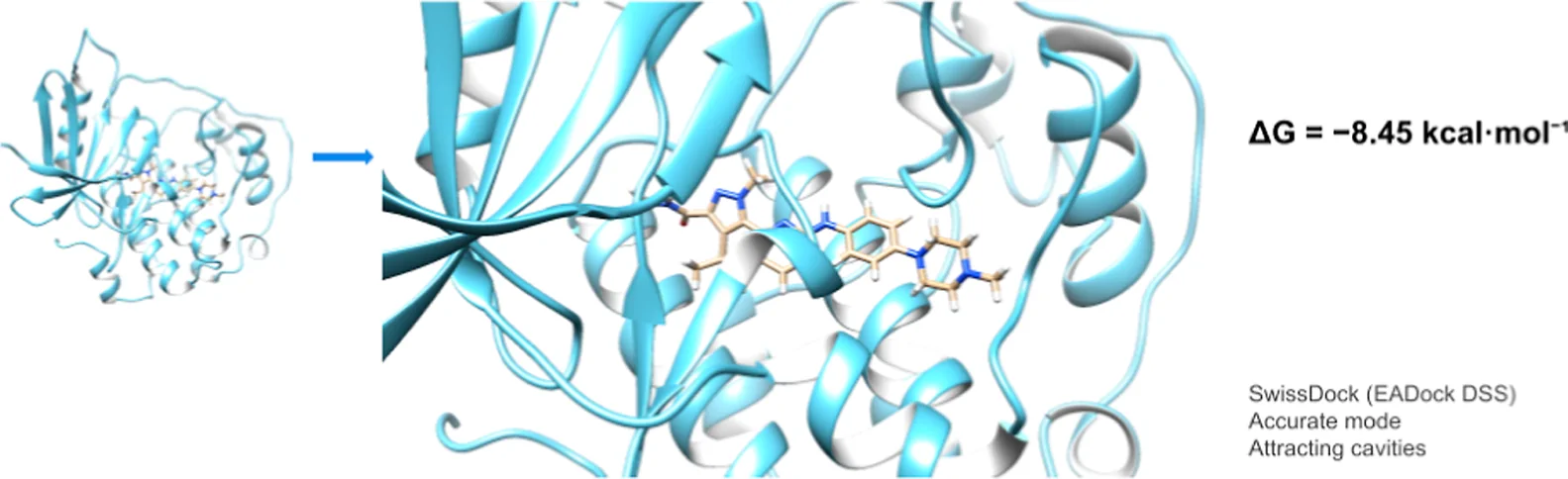
Molecular docking of MCB within the catalytic site of CRK2 from *T. gondii*. Docking analysis was performed to evaluate the interaction between MCB and CRK2. The best-ranked binding pose shows MCB positioned within the catalytic pocket, corresponding to the ATP-binding site. The ligand is accommodated within a cavity formed by α-helices and β-sheets, suggesting a stable interaction within the protein core and supporting a potential ATP-competitive inhibition mechanism.

## Discussion

Current therapeutic regimens for the treatment of congenital toxoplasmosis exhibit limited efficacy, notable toxicity, and restrictions regarding safety during pregnancy, thereby maintaining *T. gondii* infection as a significant public health challenge.^18,19^ In this context, the identification of safer and more effective therapeutic alternatives is a priority. Drug repositioning, particularly through open-access compound libraries, has been established as a valuable strategy to accelerate the discovery of novel antiparasitic candidates.^20,21^ The MMV’s Pathogen Box, provides molecules with previously described activity against diverse pathogens, including evidence of anti-T. gondii activity, highlighting the potential of this collection as a source for novel therapeutic agents.^7,8,22^

Among the compounds in this library, MCB stands out as a cyclin-dependent kinase (CDK) inhibitor initially developed for oncological applications.^9,23–25^ Its primary mechanism involves CDK inhibition, leading to cell cycle arrest and apoptosis induction.^23^ Considering that, in Apicomplexa such as *T. gondii*, cell cycle control is mediated by an expanded repertoire of CDKs orchestrating G1/S transition, DNA replication, and mitotic events in a highly specialized manner, these enzymes represent particularly attractive targets for therapeutic repositioning strategies.^11,12^ Specific members of this family perform essential functions: TgCrk2 is directly involved in G1 phase progression, whereas TgCrk4 and TgCrk6 regulate DNA replication and nuclear division, respectively, highlighting the centrality of these kinases in parasite proliferation.^11,26,27^ Although MCB’s potential has been previously explored against kinetoplastid protozoa,^13^ its investigation in the context of congenital toxoplasmosis has not yet been reported.

Docking results demonstrated that MCB is capable of binding to both parasite CRKs and human CDKs with comparable estimated binding energies. Notably, CRK2 exhibited the most favorable interaction (Δ*G* = −8.45 kcal/mol), with consistent localization of the ligand within the catalytic site. This finding suggests that CRK2 may represent a promising putative target in *T. gondii*. Taken together, these findings suggest that CRK2 may represent a structurally and functionally relevant target for MCB in *T. gondii*. Although the lack of strong energetic selectivity between parasite and host kinases represents a significant limitation for therapeutic translation, the observed differences in binding context provide a rationale for further optimization and experimental validation. This observation raises concerns regarding potential off-target effects on host CDKs, which may limit the therapeutic window and require careful optimization. Considering that MCB acts as a CDK inhibitor, a potential effect on host cells proliferation should be considered to ensure proper placental development and fetal growth. Since CDKs play essential roles in key stages of placental development, including trophoblast proliferation and differentiation,^28–30^ inhibition of host CDKs could theoretically interfere with critical physiological processes. Conversely, in this study, the cytotoxicity of MCB was evaluated in BeWo villous trophoblast cells over 72 h and in third-trimester placental villous explants, and in both models, no significant alterations in cell or tissue viability were observed compared to controls, indicating an initially favorable *in vitro* safety profile. Nevertheless, these findings should be interpreted with caution, as *in vitro* and *ex vivo* systems cannot fully reproduce the complexity of placental development or fetal growth throughout gestation. In future researches, the use of animal models will be essential to determine whether prolonged exposure to MCB can affect placental functions and embryonic development. Subsequent investigations should also focus on molecular dynamics simulations, detailed interaction mapping, and experimental assays to validate the inhibitory activity and assess selectivity profiles. Additionally, structure-based drug design approaches may be employed to improve specificity toward parasite kinases.

Subsequently, MCB exerted a robust inhibitory effect on *T. gondii* proliferation in both trophoblast cells and placental explants even at reduced concentrations and maintained persistent antiproliferative effect following drug withdrawal *in vitro*. Given that MCB significantly reduced parasite replication without compromising host cells, the potential association of this effect with modulation of the *T. gondii* cell cycle was further investigated. Analysis of cell cycle distribution demonstrated that both MCB and PYR disrupt *T. gondii* proliferative dynamics, but at distinct stages. Consistent with its antifolate mechanism, PYR reduced the G1 population while promoting accumulation in S/M, indicating that parasites enter DNA synthesis but fail to complete replication and/or division. This profile agrees with previous studies showing that inhibition of folate metabolism impairs nucleotide biosynthesis and leads to a replicative blockade during S phase,^31^ which may manifest as S/M arrest under conditions of coordinated cell cycle disruption^14^ or, alternatively, as replication collapse and loss of DNA content integrity.^31^

In contrast, MCB induced accumulation of parasites in G1 with a concomitant decrease in S/M, indicating a block prior to DNA synthesis. Notably, MCB did not affect adhesion or invasion, suggesting specificity toward intracellular replication. Given that the G1/S transition in *T. gondii* is tightly regulated by cyclin-dependent kinase-related complexes, this phenotype is consistent with the hypothesis that MCB interferes with early cell cycle regulators, possibly affecting kinase activities required for commitment to DNA synthesis.^11,12^ Similar CDK inhibitors compounds, including alvocidib, dinaciclib and SNS-032 have demonstrated potent antiparasitic activity against other protozoans, such as *T. cruzi*, *T. brucei* and *Leishmania* spp., supporting parasite CDK-related kinases as conserved therapeutic targets.^13^ Although most of these studies primarily evaluated parasite growth inhibition rather than detailed cell-cycle distribution, the antiproliferative effects were attributed to disruption of CDK-dependent regulatory pathways that regulate DNA replication and cell division, thereby supporting G1 arrest as a potential mechanistically distinct strategy to control parasite proliferation. Compared with pyrimethamine, which primarily disrupts folate metabolism and leads to S-phase arrest, MCB appears to act earlier in the cell cycle, suggesting a complementary mechanism of action that could be explored in combination therapies.

Additionally, MCB treatment induced marked structural alterations in the parasite, supporting the observed antiproliferative effect and the hypothesis of interference in essential pathways for cell cycle maintenance. Previous studies have described similar morphological changes following exposure of *T. gondii* to different drugs that impact the cell cycle progression and intracellular replication.^8,32^ In a prior report conducted by our group, *T. gondii* tachyzoites treated with other compounds from the Pathogen Box, such as MMV675968, MMV022478, and MMV021013, exhibited morphological alterations including irregular and ruptured membranes, cell body degradation, and organelle disorganization.^8^ Furthermore, Pedra-Rezende et al. (2022) reported that similar ultrastructural changes observed in different protozoan, including *T. gondii*, exposed to stress conditions or different drugs, may be associated with autophagy-like processes. Under these conditions, overactivation of autophagic pathways has been associated with impaired cellular homeostasis and reduced virulence. Although the reduction in proliferation observed in our assays may be associated with processes such as this, suggesting the induction of autophagy, the present findings do not allow definitive classification of the death phenotype as apoptosis-like, autophagic, or necrotic, since specific molecular markers were not evaluated.

Importantly, despite its direct effects on the parasite, MCB did not elicit an exacerbated inflammatory profile in trophoblast cells, with no significant increase in pro-inflammatory cytokine production. This finding is particularly relevant in the gestational context, where maternal–fetal immune balance is critical for maintaining pregnancy and ensuring appropriate fetal development.^33,34^ MCB’s ability to exert direct antiparasitic activity without triggering inflammatory dysregulation suggests a degree of pharmacological specificity in the tested models, although its safety profile for congenital infection applications remains to be established *in vivo*. In addition, although the concentrations tested *in vitro* were effective, it remains unclear whether such levels are achievable and sustainable *in vivo* without inducing systemic toxicity.

Current therapeutic regimens for congenital toxoplasmosis are based mainly on spiramycin and the pyrimethamine + sulfadiazine combination.^5,6^ Although these drugs have demonstrated clinical benefits, important limitations remain. Spiramycin is primarily effective in reducing maternal–fetal transmission, but shows limited efficacy once fetal infection is established, whereas pyrimethamine-based treatments are associated with adverse effects related to folate inhibition and are generally avoided during early pregnancy due to concerns regarding teratogenicity.^5^ In contrast, in the present study, MCB exhibited potent antiparasitic activity in both trophoblast cells and placental explants while maintaining low toxicity in maternal–fetal interface models, indicating promising potential in controlling *T. gondii* in congenital infection. Nonetheless, direct comparisons with the standard therapies should be interpreted cautiously and the safety of MCB during pregnancy remains to be established.

The activity of MCB observed in this study expands preceding findings from our group regarding Pathogen box-derived compounds evaluated in maternal–fetal interface models. In a previous study,^8^ MMV675968, MMV022478, and MMV021013 effectively controlled *T. gondii* infection in both BeWo cells and human placental explants, interfering with parasite adhesion and intracellular proliferation, while also preserving placental tissue viability. Among these compounds, MMV021013 was highlighted because of its favorable predicted pharmacokinetic and toxicity profile. Similar to these candidates, MCB owns several characteristics that support its translational potential and make it an attractive candidate for further investigation against *T. gondii*. Herein, the compound exhibited potent antiparasitic activity at low concentrations while maintaining minimal cytotoxicity in both trophoblast cells and human placental explants, resulting in a positive selectivity profile. Moreover, MCB induced a persistent inhibitory effect on parasite proliferation, interfered with *T. gondii* cell cycle progression, and preserved the local inflammatory balance at the maternal–fetal interface. These combined features are particularly relevant for congenital toxoplasmosis, since a suitable therapeutic agent should effectively control the parasitism without compromising placental integrity or disrupting the regulated immune environment required for a successful pregnancy.

In addition, MCB has been previously evaluated in oncology clinical trials, where studies conducted in cancer patients have generated valuable information regarding its pharmacokinetic properties, tolerability, dosing regimens, and safety profile.^9,23,35^ The existence of this evidence provides a more solid basis for its repositioning than is normally available for newly identified compounds and may facilitate future translational studies. Furthermore, the fact that MCB has advanced to clinical trials reinforces its pharmacological characteristics, including bioavailability and systemic exposure, which are crucial considerations for the development of antiparasitic therapies. Nevertheless, despite these advantages, its safety during pregnancy remains unknown. Additional studies are necessary to investigate maternal and fetal safety, placental transfer, optimal dosing, pharmacokinetics in gestation and long-term developmental outcomes. Furthermore, evaluation in animal models and validation of the docking analysis are essential for a better understanding of its molecular mechanism of action against *T. gondii* before considering clinical application.

## Conclusions

Collectively, the present study identifies MCB as a promising candidate for drug repositioning against congenital toxoplasmosis. Beyond its potent antiparasitic activity and low toxicity in human trophoblast cells and placental villi, MCB interfered with *T. gondii* cell cycle progression, preventing the parasite proliferation without enhancing the inflammatory response, maintaining the maternal–fetal interface homeostasis. Coupled with *in silico* evidence indicating its interaction with parasite CDKs, these data provide a consistent rational for further investigations, supporting the potential of MCB as a candidate for the development of safer treatment approaches. Additional data regarding pharmacological properties and performance in *in vivo* models are needed to fully establish MCB as a therapeutic potential for use during pregnancy, and future studies should be undertaken accordingly.

## Methods

### Maintenance of Cell Cultures and Parasite

BeWo cells (ATCC, CCL-98, Manassas, VA), a human villous trophoblast cell line, were used as the representative trophoblast model. Cells were cultured in RPMI 1640 medium (Cultilab, Campinas, SP, Brazil) supplemented with 100 U/mL penicillin (Sigma Chemical Co., St. Louis, MO), 100 μg/mL streptomycin (Sigma), and 10% heat-inactivated fetal bovine serum (FBS) (Cultilab) in a humidified incubator at 37 °C with 5% CO_2_.

*T. gondii* tachyzoites from the RH strain expressing the β-galactosidase reporter gene were used in this study, allowing precise quantification of parasite burden via a colorimetric enzymatic assay based on the hydrolysis of the chromogenic substrate chlorophenol red-β-d-galactopyranoside (CPRG). Parasites were propagated through serial passage in BeWo cells cultured in RPMI 1640 medium supplemented with penicillin, streptomycin, and 2% FBS at 37 °C with 5% CO_2_.

### Chemical Compound

MCB (MMV676602) from the Pathogen Box was obtained by the Medicines for Malaria Venture (MMV). The compound was dissolved in dimethyl sulfoxide (DMSO) to prepare a 10 mM stock solution and then diluted in RPMI 1640 supplemented with FBS before use. The final concentration of DMSO in all treatments was maintained below 0.1%, a level that is noncytotoxic to BeWo cells.^8^

### Human Placenta Collection

Human placental explants were used as an *ex vivo* model of the maternal–fetal interface. Placentas were collected from cesarean deliveries performed electively (36–40 weeks of gestation) at the Clinical Hospital of the Universidade Federal de Uberlândia (HC-UFU), Minas Gerais, Brazil. Donor selection criteria excluded patients with comorbidities, including chronic hypertension, pre-eclampsia, diabetes, or infectious diseases such as toxoplasmosis, Chagas disease, and leishmaniasis. Only donors between 18 and 45 years of age were eligible for tissue collection. Following delivery, placentas were washed with phosphate-buffered saline (PBS) to remove excess maternal blood. Chorionic villous explants containing approximately four to eight free tips (∼10 mm^3^) were collected. To allow tissue equilibration, explants were incubated in RPMI 1640 supplemented with 10% FBS for 24 h (h) at 37 °C with 5% CO_2_ prior to subsequent assays.^35^ This study was conducted in accordance with protocols approved by the Ethics Committee of the Universidade Federal de Uberlândia (UFU), Minas Gerais, Brazil (approval number 7407162).

### Cell Viability Assays

To identify safe concentrations for use in the cell model, a colorimetric assay based on 3-(4,5-dimethylthiazol-2-yl)-2,5-diphenyltetrazolium bromide (MTT) was employed.^8^ Briefly, BeWo cells were seeded at a density of 1 × 10^4^ cells/well in 96-well plates and cultured for 24 h in RPMI 1640 medium supplemented with 10% FBS at 37 °C with 5% CO_2_, and then exposed for 72 h to three serially diluted concentrations of MCB (0.40, 0.81, and 1.62 μM; 2-fold dilutions). The tested concentrations were selected based on the IC_50_ value (0.81 μM) reported by Spalenka et al.,^7^ as well as on concentrations corresponding to half (0.40 μM) and twice (1.62 μM) this IC_50_, to evaluate dose-dependent effects across a biological range. Cells incubated with RPMI 1640 medium alone served as the negative control and were defined as 100% viability. Additionally, cells were exposed to pyrimethamine (PYR; Sigma), a first-line drug for toxoplasmosis, at a concentration of 1 μM.^8^ After the treatment period, supernatants were removed and an MTT solution (5 mg/mL in RPMI medium, Sigma) was added for 4 h at 37 °C with 5% CO_2_. Formazan crystals formed by viable cells were solubilized in 10% SDS and 50% *N*,*N*-dimethylformamide for 4 h, and absorbance was measured at 570 nm using a plate reader. Cell viability was expressed as the percentage of viable cells compared with the untreated control group.

### *T. gondii* Proliferation Assays

The potential of MCB to interfere with *T. gondii* proliferation in BeWo cells was evaluated by a β-galactosidase enzymatic assay, following previously established protocols.^36^ Briefly, BeWo cells were seeded (1 × 10^4^ cells/well) in 96-well plates for 24 h and subsequently infected with *T. gondii* tachyzoites at a multiplicity of infection (MOI) of 1:1 (one parasite per cell) for 3 h. Noninternalized parasites were removed by washing the wells with PBS. Infected BeWo cells were then treated with MCB at concentrations of 0.40, 0.81, and 1.62 μM for 72 h at 37 °C with 5% CO_2_. PYR (1 μM) and RPMI 1640 medium alone were used as positive and negative controls, respectively. Parasite burden was assessed by β-galactosidase assay upon addition of the chromogenic substrate CPRG (Roche Diagnostics, Mannheim, Germany), and absorbance was read at 570 nm. A standard curve of free tachyzoites (ranging from 1 × 10^6^ to 1.56 × 10^4^ parasites) was used to quantify parasite numbers.

### Reversibility Assays

The potential reversibility of *T. gondii* proliferative capacity in MCB-treated BeWo cells was assessed following previously described methods.^36^ BeWo cells were seeded (1 × 10^4^ cells/well) in 96-well plates for 24 h in RPMI 1640 medium supplemented with 10% FBS at 37 °C with 5% CO_2_. Subsequently, cells were infected with *T. gondii* tachyzoites at an MOI of 1:1 for 3 h at 37 °C with 5% CO_2_, after which wells were washed with PBS to remove noninternalized parasites. Infected cells were subsequently treated with MCB (0.81 μM), PYR (1 μM), or culture medium alone. Following the treatment period, parasite burden was either assessed immediately by β-galactosidase assay, or cells were washed with PBS and incubated in RPMI medium alone for another 24 h, after which *T. gondii* proliferation was quantified. Parasite proliferation was expressed as a percentage, with the medium-only group defined as 100% proliferation.

### Cell Cycle Analysis

*T. gondii* cell cycle progression was evaluated by flow cytometry following previously published protocols^36–38^ with minor adjustments. BeWo cells were seeded in 6-well plates for 24 h to allow adhesion, and then infected with *T. gondii* tachyzoites at an MOI of 1:1 in supplemented RPMI 1640 medium for 3 h at 37 °C with 5% CO_2_. Infected cells were then exposed to MCB (0.81 μM), PYR (1 μM), or culture medium alone for 72 h at 37 °C with 5% CO_2_. Intracellular parasites were subsequently recovered by serial passages through 21- and 26-gauge needles, washed with PBS, and fixed overnight in 70% ethanol at 4 °C. Fixed parasites were incubated for 16 h with a mouse monoclonal anti-*T. gondii* antibody targeting SAG1/p30 (clone TP3, Abcam, #ab8313) diluted in PBS. Following a PBS wash, parasites were incubated with Alexa Fluor 488-conjugated antimouse IgG (Invitrogen, #A11001), RNase A (100 μg/mL), and propidium iodide (PI, 10 μg/mL), all prepared in PBS. Staining was performed for 45 min in the dark at room temperature. DNA content was measured on a CytoFLEX flow cytometer (Beckman Coulter), and data were analyzed using FlowJo v.7.6.

### Invasion and Proliferation Assays with Pretreated Parasites

To assess the capacity of MCB to interfere with initial steps of *T. gondii* infection in BeWo cells, parasite invasion was evaluated following pretreatment of tachyzoites with the compound.^36^ Briefly, *T. gondii* tachyzoites were incubated for 1 h with MCB (0.81 μM), PYR (1 μM), or culture medium alone, and subsequently allowed to infect BeWo cells previously seeded in 96-well plates at an MOI of 1:1 for 3 h at 37 °C with 5% CO_2_. After the invasion period, noninternalized parasites were removed by PBS washing, and internalized parasites were quantified by β-galactosidase assay.

For the proliferation assay, *T. gondii* tachyzoites were treated as described in the paragraph above. Following the pretreatment, the parasites were incubated for 72 h with BeWo cells previously seeded in 96-well plates at an MOI of 1:1 for 3 h at 37 °C with 5% CO_2_. After this period, *T. gondii* proliferation was measured through β-galactosidase assay. Both invasion and proliferation were expressed as percentages, with the medium-only group defined as 100% invasion or proliferation, respectively.

### Adhesion Assays with Pretreated Parasites

To complement the results obtained from the invasion and proliferation assays of previously treated *T. gondii*, we conducted parasite adhesion assays in BeWo cells following previously established protocols.^14,36^ *T. gondii* tachyzoites were pretreated with MCB (0.81 μM), PYR (1 μM), or culture medium alone for 1 h at 37 °C with 5% CO_2_. Subsequently, pretreated parasites were incubated for 3 h with BeWo cells previously fixed with 4% paraformaldehyde (PFA) in glass coverslips (13 mm, in 24-well plates) for 30 min at room temperature. Following PBS washing to remove nonadherent tachyzoites, the remaining parasites were fixed under the same conditions described for BeWo cells. Coverslips were subsequently incubated with a rabbit polyclonal primary anti-*T. gondii* antibody (Abcam #20530; Waltham, MA) (diluted 1:500 in PGN-0.01% saponin solution) for 1 h, followed by staining with TO-PRO-3 Iodide (1:500) to visualize nuclei, TRITC-conjugated phalloidin (1:50) to label F-actin, and Alexa Fluor 488-conjugated antirabbit IgG (1:500) to identify tachyzoites. Fluorescence imaging was performed by confocal microscopy (Zeiss LSM 510 Meta, Germany). Alexa Fluor 488 was excited at 488 nm using an argon laser, TRITC at 543 nm using a HeNe laser, and TO-PRO-3 at 633 nm using a HeNe laser; emission signals were collected at 519, 570, and 661 nm, respectively. A minimum of 20 randomly selected fields were analyzed to determine the total number of tachyzoites associated with host cells per field. Assays with pretreated BeWo cells.

To determine whether prior exposure of BeWo cells to MCB modulates the capacity of *T. gondii* to adhere, invade, and proliferate to host cells, trophoblastic cells were pretreated with MCB prior to parasite challenge.^37^ Two approaches were used for this analysis: quantification of *T. gondii* by β-galactosidase assay and by immunofluorescence.

Briefly, BeWo cells were cultured in 96-well plates overnight and then incubated for 72 h with MCB (0.81 μM), PYR (1 μM), or culture medium alone at 37 °C with 5% CO_2_. Pretreated cells were then infected with *T. gondii* tachyzoites (MOI 1:1) for either 3 or 72 h to assess invasion or proliferation, respectively. Parasite burden was quantified by β-galactosidase assay and presented as a percentage, with the medium-only group defined as 100% invasion or proliferation.

For immunofluorescence analysis, BeWo cells were seeded on glass coverslips (13 mm, in 24-well plates) for 24 h at 37 °C with 5% CO_2_, then treated with MCB (0.81 μM), PYR (1 μM), or culture medium alone for 72 h at 37 °C with 5% CO_2_. Following treatment, cells were infected by *T. gondii* tachyzoites (MOI 1:1) for 3 h at 37 °C and 5% CO_2_. Subsequently, the coverslips were washed with PBS and fixed with 4% PFA for 30 min at room temperature. Cells and parasites were then labeled with TO-PRO-3 Iodide (1:500; nuclei), Alexa Fluor 488-conjugated antirabbit IgG (1:500; internalized parasites), and Alexa Fluor 594-conjugated antirabbit IgG (1:500; adherent parasites). Fluorescence images were acquired using confocal microscopy (Zeiss LSM 510 Meta, Germany) using the same acquisition parameters described above. Intracellular (Alexa Fluor 488^+^/Alexa Fluor 594^–^) and adherent [Alexa Fluor 594^+^, or Alexa Fluor 488^+^/Alexa Fluor 594^+^ (yellow)] parasites were quantified across a minimum of 20 randomly selected fields per coverslip, and results were described as the number of tachyzoites per field.

### Transmission Electron Microscopy (TEM)

To provide additional insight into the effects of MCB on *T. gondii*, potential morphological alterations in intracellular parasites were examined in MCB-treated BeWo cells by transmission electron microscopy (TEM) following previous studies.^8^ BeWo cells were seeded in 6-well plates for 24 h, infected with *T. gondii* tachyzoites (MOI 1:1) for 3 h, washed with PBS, and subsequently incubated for 72 h with MCB (0.81 μM), PYR (1 μM), or culture medium alone at 37 °C with 5% CO_2_. Cells were then washed with PBS, collected, and fixed in Karnovsky’s solution (2% PFA and 2% glutaraldehyde in 0.1 M sodium cacodylate buffer, pH 7.4) for 24 h at 4 °C. Subsequently, samples were incubated for 1 h in 1% osmium tetroxide (OsO_4_) in cacodylate buffer, treated with potassium ferrocyanide (K_4_[Fe­(CN)_6_]) for 30 min, and embedded in agar. Dehydration was carried out through a graded ethanol series (50, 70, 80, 90, 95, and 100%) followed by propylene oxide, and samples were embedded in Epon resin. Ultrathin sections were subsequently contrasted with uranyl acetate and lead citrate before analysis by transmission electron microscopy (Hitachi HT7700).

### Evaluation of Reactive Oxygen Species (ROS) Production

To investigate possible mechanisms underlying MCB activity against *T. gondii* infection in BeWo cells, reactive oxygen species (ROS) production was assessed.^39,40^ Briefly, BeWo cells were cultured in 96-well plates overnight and then infected or not with *T. gondii* tachyzoites (MOI 1:1) for 3 h at 37 °C with 5% CO_2_. Noninternalized parasites were then removed using PBS, and cells were incubated for 72 h with MCB (0.81 μM), PYR (1 μM), or culture medium alone at 37 °C with 5% CO_2_. After the treatment period, cells were washed with PBS and incubated with 2′,7′-dichlorodihydrofluorescein diacetate (H_2_DCF-DA, Invitrogen, catalog number D399) at a concentration of 10 μM diluted in 1× PBS with 10% FBS for 45 min at 37 °C with 5% CO_2_ in the dark. Fluorescence was measured using a spectrofluorometer (VersaMax, Molecular Devices, CA) at excitation/emission wavelengths of 488/522 nm. Data were described as median fluorescence intensity (MFI).

### Assessment of Human Placental Villous Explants Viability

Explant viability was evaluated by MTT assay and lactate dehydrogenase (LDH) quantification following established protocols.^14,37^ Placental explants were treated with MCB (0.81 μM), PYR (1 μM), or culture medium alone (untreated control, defined as 100% viability) for 72 h. After the treatment period, culture supernatants were collected for LDH quantification according to the manufacturer’s kit instructions (LDH UV, Bioclin, Belo Horizonte, MG, Brazil|ref K014-2), with absorbance measured at 340 nm. In parallel, for the MTT assay, explants were incubated with MTT (5 mg/mL) for 4 h at 37 °C with 5% CO_2_. Formazan crystals were then solubilized in 10% SDS and 50% *N*,*N*-dimethylformamide for 18 h at 37 °C with 5% CO_2_. Villi were removed, and absorbance of the remaining culture medium was measured at 570 nm. As a complementary qualitative assessment, treated explants were processed for histological analysis by hematoxylin and eosin (H&E) staining and analyzed by light microscopy to evaluate placental tissue morphology.

### Evaluation of *T. gondii* Proliferation in Human Placental Explants

To assess the capacity of MCB to interfere with *T. gondii* infection in the *ex vivo* model, placental explants were incubated with *T. gondii* tachyzoites (1 × 10^6^ per explant) overnight at 37 °C with 5% CO_2_. Following, explant cultures were washed with PBS to remove noninternalized parasites and subsequently treated with MCB (0.81 μM), PYR (1 μM), or culture medium only for 72 h at 37 °C with 5% CO_2_. Explants were then collected and stored at −80 °C until further analysis.^36^

Parasite burden in placental explants was quantified by β-galactosidase assay. In brief, frozen villi were disrupted by liquid nitrogen-induced freezing and macerated in radioimmunoprecipitation assay (RIPA) buffer [50 mM Tris–HCl, 150 mM NaCl, 1% Triton X-100, 1% sodium deoxycholate, and 0.1% SDS, pH 7.5] containing a protease inhibitor cocktail (cOmplete, Roche Diagnostics, Mannheim, Germany). The homogenate was centrifuged (21,000*g* for 15 min at 4 °C), and the resulting supernatant was used for the β-galactosidase assay as described above. Results were normalized according to total protein content, quantified by the Bradford method: Bradford reagent was added to the supernatant in 96-well plates, and absorbance was measured at 595 nm. Data are expressed as the number of parasites per microgram of total protein (μg/mL).

### Cytokine Quantification

To further elucidate the mechanisms underlying MCB activity against *T. gondii* infection in both the *in vitro* (BeWo cells) and *ex vivo* (human placental explants) models, cytokines of relevance to the infectious context at the maternal–fetal interface were quantified by enzyme-linked immunosorbent assay (ELISA), following the manufacturers’ protocols. IL-6, IL-8, and TNF were measured using OptEIA kits (BD Biosciences, San Diego, CA), and MIF was quantified through a DuoSet kit (R&D Systems, Minneapolis, MN). For placental explant supernatants, cytokine concentrations were normalized to total protein content as determined by the Bradford method described above, and results were expressed as pg/mg of tissue. For BeWo cells, results were expressed as pg/mL. The lower detection limits, as determined from standard curves, were 4.7 pg/mL for IL-6, 3.1 pg/mL for IL-8, 7.8 pg/mL for MIF, and 7.8 pg/mL for TNF.

### Molecular Docking

Human cyclin-dependent kinases CDK1 and CDK4 were selected as reference sequences due to their known interaction with MCB. Protein sequences were retrieved from public databases and used as queries in BLASTp searches against the *T. gondii* proteome available in ToxoDB. Candidate targets were selected based on the following criteria: significant *e*-values, moderate sequence identity (30–50%), and adequate coverage of the kinase catalytic domain. Due to evolutionary divergence, the identified proteins corresponded to CDK-related kinases (CRKs), rather than direct orthologs of human CDKs. Three candidate targets were selected for further analysis: CRK1 (TGME49_304970), CRK2 (TGME49_218220), and CRK3 (TGME49_285160), prioritized based on domain conservation and structural features.

The chemical structure of MCB (PubChem CID: 16718576) was obtained from the PubChem database. The ligand was submitted to SwissDock using its SMILES representation, allowing automatic 3D structure generation, protonation, and conformational sampling. Molecular docking simulations were performed using the “Accurate” mode. A blind docking approach was applied to avoid bias in binding site selection.^41,42^ Both *T. gondii* CRKs and human CDK1 and CDK4 were used as receptors.

### Statistical Analysis

All statistical analyses and graphs were generated using GraphPad Prism 8.0 (GraphPad Software, Inc., San Diego, CA). Results are reported as mean ± standard error of the mean (SEM). Comparisons across multiple groups were assessed using one-way ANOVA with Šídák’s multiple comparison posttest when data met parametric assumptions; otherwise, the Kruskal–Wallis test with Dunn’s post hoc correction was applied.^43,44^ A *p* value < 0.05 was considered statistically significant. All results reflect at least three independent experiments, each carried out with a minimum of eight replicates.

## Supplementary Material



## Data Availability

The data in the current study are available from the corresponding author upon request.

## Notes

Table S1, molecular docking results of MCB against *T. gondii* CRKs and human CDKs; Table S2, identification of CDK-related kinases in *T. gondii* by sequence similarity analysis; and Figure S1, domain organization of *T. gondii* CRK2 (PDF)

## Abbreviations

MCB: milciclib
MIF: macrophage migration inhibitory factor
ROS: reactive oxygen species
MMV Pathogen Box: Medicines for Malaria Venture Pathogen Box
FBS: fetal bovine serum
CPRG: chlorophenol red-β-d-galactopyranoside
DMSO: dimethyl sulfoxide
HC-UFU: Clinical Hospital of the Universidade Federal de Uberlândia
PYR: pyrimethamine
TEM: transmission electron microscopy
LDH: lactate dehydrogenase
RIPA: radioimmunoprecipitation assay buffer
ELISA: enzyme-linked immunosorbent assay
CDKs: cyclin-dependent kinases
