# Supplementary material for: Repurposing of Milciclib as a Potential Inhibitor of Toxoplasma gondii in Human Trophoblasts and Placental Explants
Source: ACS Omega. 2026 Sep 28;11(40):61168–82. doi: 10.1021/acsomega.6c05747 (PMC13670761; doi:10.1021/acsomega.6c05747)

Repurposing of Milciclib as a potential inhibitor of *Toxoplasma gondii* in human trophoblasts and placental explants

Rafael Martins de Oliveira[1], Natália Carine Lima dos Santos[1], Joed Pires de Lima Júnior[1], Luana Carvalho Luz[1], Marcos Paulo Oliveira Almeida[1], Guilherme de Souza[1], Izadora Santos Damasceno[1], Muriel Pereira Souto[1], Rosiane Nascimento Alves[2,3], Angelica Oliveira Gomes[4], Ingrid de Oliveira Dias[5], Juliana Quero Reimão[5], Bellisa Freitas Barbosa[1], Eloisa Amália Vieira Ferro[1] and Samuel Cota Teixeira[1*]

[1]Laboratory of Immunophysiology of Reproduction, Institute of Biomedical Science, Universidade Federal de Uberlândia, Uberlândia, MG, Brazil;
[2]Department of Agricultural and Natural Science, Universidade do Estado de Minas Gerais, Ituiutaba, MG, Brazil;
[3]Departament of clinical medicine, Medicine College, Universidade Federal de Uberlândia, Minas Gerais, Brazil;
[4]Laboratory of Cell Interactions, Institute of Natural and Biological Sciences, Universidade Federal do Triângulo Mineiro, Uberaba, MG, Brazil;
[5]Laboratory of Preclinical Assays and Research of Alternative Sources of Innovative Therapy for Toxoplasmosis and Other Sicknesses (Lab. PARASITTOS), Department of Morphology and Basic Pathology, Faculdade de Medicina de Jundiaí, Jundiaí, SP, Brazil.

*Corresponding author:
Dr. Samuel Cota Teixeira
Institute of Biomedical Science
Universidade Federal de Uberlândia, Campus Umuarama, Av. Pará, 1720 | 38405318
Uberlândia-MG, Brazil
Phone: +55 34 3225-8670/3239-1363
E-mail address: samuel.teixeira@ufu.br

Supporting information

Table S1. Molecular docking results of MCB against *Toxoplasma gondii* CRKs and human CDKs. Estimated binding free energies (ΔG, kcal/mol) obtained from molecular docking simulations are presented for MCB in complex with *T. gondii* CRK1, CRK2, and CRK3, as well as human CDK1 and CDK4. The values indicate comparable binding affinities across targets, with CRK2 showing the most favorable interaction.

Protein	ΔG (kcal/mol)	Binding Site
CRK1 (*T. gondii*)	−7.22	Catalytic site
CRK2 (*T. gondii*)	−8.45	Catalytic site
CRK3 (*T. gondii*)	−7.64	Undefined
CDK1 (Human)	−7.33	ATP-binding pocket
CDK4 (Human)	−7.41	ATP-binding pocket

Table S2. Identification of CDK-related kinases in *Toxoplasma gondii* by sequence similarity analysis. BLASTp analysis was performed using human CDK sequences as queries against the *T. gondii* proteome. Candidate CDK-related kinases (CRKs) were identified based on e-values, sequence identity, and domain coverage. The table summarizes alignment metrics supporting the selection of CRK1, CRK2, and CRK3 for further structural and docking analyses.

Query (Human CDK)	Target (*T. gondii*)	Gene ID	Annotation	E-value	Priority
CDK1	CRK2	TGME49_218220	CDK-related kinase	5e-125	High
CDK1	CRK1	TGME49_304970	CDK-related kinase	5e-71	Medium
CDK1	CRK3	TGME49_285160	CDK family protein	2e-65	Low
CDK4	CRK2	TGME49_218220	CDK-related kinase	5e-115	High
CDK4	CRK1	TGME49_304970	CDK-related kinase	4e-81	Medium
CDK4	CRK3	TGME49_285160	CDK family protein	2e-70	Low

Figure S1. Domain organization of CRK2 from *Toxoplasma gondii*. The domain architecture of CRK2 was analyzed using Pfam/InterPro. The protein is predominantly composed of a conserved kinase domain spanning most of its sequence, consistent with canonical cyclin-dependent kinase-related proteins and supporting its functional annotation.

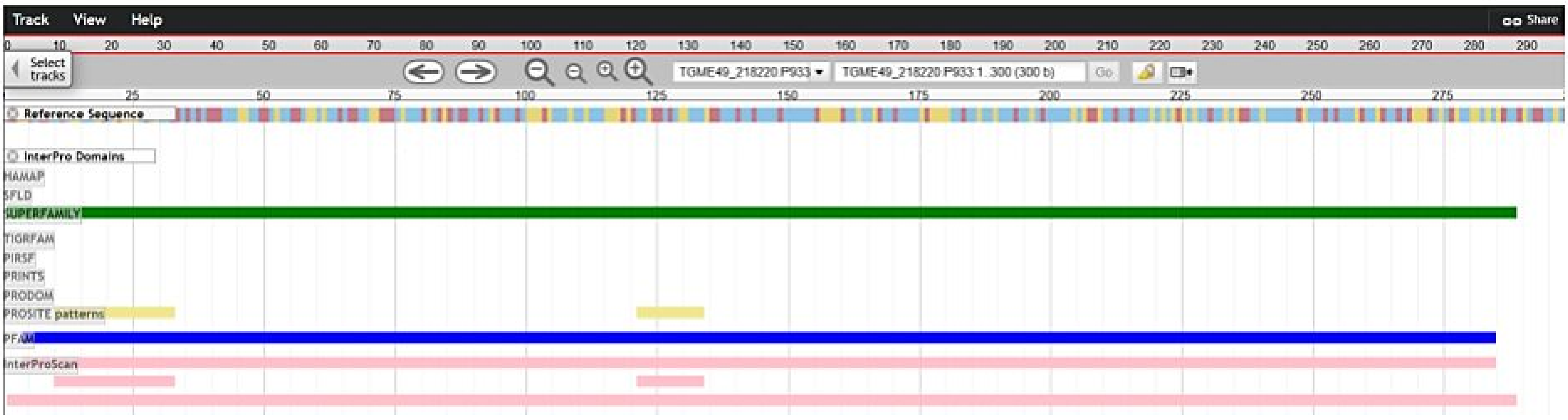

Supplement: Supplementary file 1 [file ao6c05747_si_004.pdf]
